# Flavonoid-rich orange juice is associated with acute improvements in cognitive function in healthy middle-aged males

**DOI:** 10.1007/s00394-015-1016-9

**Published:** 2015-08-18

**Authors:** Mudi H. Alharbi, Daniel J. Lamport, Georgina F. Dodd, Caroline Saunders, Laura Harkness, Laurie T. Butler, Jeremy P. E. Spencer

**Affiliations:** 1 School of Psychology and Clinical Language Sciences, University of Reading, Reading, RG6 6AL UK; 2 Molecular Nutrition Group, School of Chemistry, Food and Pharmacy, University of Reading, Reading, RG6 6AP UK; 3 PepsiCo Inc., Reading, UK

**Keywords:** Flavonoids, Flavanones, Cognition, Cognitive function, Orange juice

## Abstract

**Purpose:**

Epidemiological evidence suggests that chronic consumption of fruit-based flavonoids is associated with cognitive benefits; however, the acute effects of flavonoid-rich (FR) drinks on cognitive function in the immediate postprandial period require examination. The objective was to investigate whether consumption of FR orange juice is associated with acute cognitive benefits over 6 h in healthy middle-aged adults.

**Methods:**

Males aged 30–65 consumed a 240-ml FR orange juice (272 mg) and a calorie-matched placebo in a randomized, double-blind, counterbalanced order on 2 days separated by a 2-week washout. Cognitive function and subjective mood were assessed at baseline (prior to drink consumption) and 2 and 6 h post consumption. The cognitive battery included eight individual cognitive tests. A standardized breakfast was consumed prior to the baseline measures, and a standardized lunch was consumed 3 h post-drink consumption.

**Results:**

Change from baseline analysis revealed that performance on tests of executive function and psychomotor speed was significantly better following the FR drink compared to the placebo. The effects of objective cognitive function were supported by significant benefits for subjective alertness following the FR drink relative to the placebo.

**Conclusions:**

These data demonstrate that consumption of FR orange juice can acutely enhance objective and subjective cognition over the course of 6 h in healthy middle-aged adults.

## Introduction

Epidemiological data suggest that frequent consumption of citrus fruits is associated with various health benefits such as a reduced risk of cardiovascular disease, cerebral infarction and ischemic stroke [1–3]. Recently, there has also been interest in the relationship between fruit consumption and cognitive function [4], particularly given that fruits and juices provide a rich, easily available source of flavonoids such as hesperidin and narirutin. Human research of this field is in its infancy; however, recent reviews indicate that increased flavonoid consumption over the lifespan may attenuate age-associated cognitive decline and the onset of neurodegenerative disease [5, 6]. For example, increased consumption of citrus fruits and orange juice was associated with better cognitive outcomes in a cross-sectional analysis of 2031 females aged 70–74 [7].

In support of the epidemiological evidence, a handful of intervention studies in older adults with mild cognitive impairment indicate that daily intake of flavonoid-rich (FR) juices over 12–16 weeks can benefit memory function [8–10]. Cognitive benefits following chronic fruit juice consumption are not exclusive to adults with mild cognitive impairment; improvements in global cognitive function were observed in healthy older adults (mean age 67) following 8-week daily consumption of flavanone-rich orange juice (305 mg/day) relative to a low-flavanone control (37 mg) [11]. It has been hypothesized that increased cerebral blood flow (CBF) and increased neural activity following consumption of flavonoid-rich drinks could provide an underlying mechanism for the aforementioned cognitive benefits [9, 12]. In support of this, daily dietary supplementation of cocoa flavanols (900 mg/day) over 8 weeks was associated with increased cerebral blood volume in the dentate gyrus of the hippocampus [13]. Crucially, concomitant improvements in pattern recognition were also observed, such that response times were 630 ms quicker following high-flavanol relative to low-flavanol supplementation. This provides evidence of possible causation between flavonoid intake, increased CBF and behavioral effects.

It remains to be seen whether acute cognitive benefits occur within the immediate postprandial phase following juice intake. However, acute improvements in memory and executive function have been observed in healthy young adults following consumption of flavonoid-rich cocoa-based drinks and solids [14, 15]. The aim of the present research was to investigate whether consumption of FR orange juice is associated with acute cognitive benefits in healthy middle-aged adults. Orange juice is one of the most commonly consumed sources of flavonoids, and the potential for fruits and fruit-based drinks to enhance cognitive function or attenuate cognitive decline deserves investigation, particularly in healthy populations for whom very limited published data exist. The flavonoid concentration of whole oranges can be higher than the concentration found in orange juice [16], principally because the fibers in orange act as an entrapping matrix [17]; thus, some of the flavonoids are lost during the juicing process. Therefore, in the present study, the intervention drink is composed of whole processed juicing orange which retains the fiber, and thus delivers a high dose of flavonoids.

## Subjects and methods

### Participants

Twenty-four healthy males were recruited from Reading, UK, and surrounding areas. Two participants withdrew following the completion of arm one due to work commitments (data not included). Inclusion criteria were males aged 30–65 (mean 51, SD 6.6), native English speaking, BMI 25–32 kg/m^2^ (mean 28.3, SD 3.1), non-smokers. Exclusion criteria were history of stroke/myocardial infarction, alcohol misuse, renal or bowel disease, pancreatitis, diabetes or any other endocrine disorder, dementia or mild cognitive impairment (mini–mental state examination [18] (MMSE) <26), current weight loss regime, vegetarian diet, regular intake of supplements including fish oil, fatty acid, vitamins and minerals, any medication for hypertension, hyperlipidaemia, inflammation, hypercholesterolemia and hypercoagulation, and history or evidence of depression (as indicated by the Brief Symptom Inventory, BSI). Pilot data by our group with a sample size of 24 healthy adults using the Digit Symbol Substitution Test (DSST) indicated an effect size (eta squared) of 0.32 following consumption of a fruit-based juice. Therefore, for a crossover design, assuming two-sided test criteria with alpha level 0.05 and a correlation of 0.5 between repeated-measures test scores, for two treatment conditions and three time-point measurements, a sample size of 24 would give power of at least 86 %. Recruitment commenced in June 2012 and terminated in December 2012.

### Design

A randomized, double-blind, placebo-controlled, crossover design was used, and good clinical practice and ethical principles were followed according to ICH and the Declaration of Helsinki (1996). There were two drink conditions: placebo and FR orange juice, and measurements were taken at three time points: baseline (prior drink consumption), 2 h post consumption and 6 h post consumption of the drink. All procedures were approved by the University of Reading Research Ethics committee (No. 12/06).

### Treatment drinks

The 240-ml FR drink consisted of Tropicana Pure Premium Orange Juice with 5.5 g of added orange pomace fiber (240 ml) which contained a total of 272 mg flavonoids (see Table 1 for drink composition). Analysis of the flavonoid content was performed by PepsiCo (Barrington, Illinois, USA). The orange pomace comprised of the micronized, edible part of the whole orange which is leftover during the production of Tropicana Pure Premium Orange Juice. The pomace is rich in fiber (40:60 ratio of soluble to insoluble) and contains small amounts of micronutrients, and a high proportion of the flavonoids found in whole orange. The control drink was prepared by dissolving a placebo mix containing glucose, fructose, sucrose and 0.67 % citric acid (for flavor purposes) in 240 ml of water. The drinks were closely matched for volume, taste, appearance, energy, glucose, fructose and sucrose (see Table 1). The ingredients for both drinks (except water) were provided by PepsiCo Inc. and were kept frozen (−20 °C) until preparation. The drinks and randomization sequence were prepared at the University of Reading by an independent researcher (AW) who did not come into contact with the participants or administer any of the test-day procedures. Both drinks were stored frozen (−20 °C) as individual portions (240 ml) in aluminum canisters and labeled with a three-digit code known only by AW. Blinded experimenters (MA, GD) performed the test-day procedures and provided participants with the drinks. Participants consumed the drinks from the canisters with black straws. The key for the codes remained sealed until data analysis was complete.

**Table 1:** Nutritional composition of the high-flavonoid and placebo drinks per 240 ml serving

	Flavonoid rich	Placebo
Energy (kcal)	92.8	87.7
Glucose (g)	5.1	5.36
Fructose (g)	6.12	6.38
Sucrose (g)	11.99	10.2
Fiber (g)	5.50	–
Vitamin C (mg)	80.17	–
Folate (mg)	65.28	–
Total B-carotenes (mg)	0.26	–
Hesperidin (mg)	220.46	–
Narirutin (mg)	34.54	–
Other flavonoids^a^ (mg)	17.14	–
Total flavonoids (mg)	272.14	–

^a^Includes didymin, sinensetin, nobiletin, tetramethylscutellarein and tangeretin

### Procedure

Participants initially attended a screening visit during which inclusion and exclusion criteria were checked with the MMSE [18] (mean 28.2, SD 1.5), BSI (mean 1.05, SD 1.89) and a Health and Lifestyle Questionnaire. A practice version of the cognitive battery was completed for familiarization (data not collected). In addition, participants also completed the following tests which provide a proxy for IQ, National Adult Reading Test (NART; mean score 30.5, SD 8.5) and Block Design (from the Wechsler Adults Intelligence Scale-R [19]; mean score 38.2, SD 9.2). Height (mean 1.8 m, SD 7 cm) and weight (92.8 kg, SD 3.1) were also measured. During screening, participants were provided with oral and typed instructions to consume a low-polyphenol diet 24 h prior to each test day which included a description of foods and drinks to avoid (berries, fruits, fruit juices, jams and preserves, red wine, black, green and fruit teas, coffee, cocoa, soy products, caffeinated energy drinks and vegetables except potatoes). The screening visit and each test day were separated by a 2-week washout. The evening prior to each test-day participants consumed (at home) a low-fat standardized chicken and rice meal provided by the research team (350 kcal, 6.9 g fat of which 3 g saturates, 52.1 g carbohydrate of which 9.7 g sugars, 19 g protein, 1.4 g fiber, 0.9 g salt) to avoid second meal cognitive effects [20]. For each test day, participants arrived at 8:30 a.m. following a 12-h fast. During the fast, participants were permitted to consume only water provided at screening. A standardized breakfast of croissants and low-fat cream cheese was consumed upon arrival (24.5 g fat, 10.1 g protein, 38.4 g carbohydrates, 415 kcal). The baseline cognitive battery commenced at 9:10 a.m. followed by the consumption of the FR or control drink at 10:00 a.m. Instructions were to consume the whole drink within 5 min. Repeated testing of the cognitive battery commenced 2 and 6 h post-drink consumption (approximately 12:00 and 16:00 h, respectively). Following completion of the second cognitive battery, a lunch was provided (identical to breakfast at approximately 13:00). The rationale for cognitive testing at 2 and 6 h was based on the predicted bioavailability of hesperidin and narirutin metabolites in the plasma which peak between 4 and 7 h postingestion [21, 22]. All visits took place at the University of Reading Hugh Sinclair Nutrition Unit. Participants remained within the Nutrition Unit for the entire test day during which only water consumption was permitted (notwithstanding the test-day foods and drinks). Participants received a £100 honorarium upon completion of both arms.

### Cognitive battery and subjective mood

The 45-min cognitive battery consisted of the following tests administered in the respective order: Immediate Word Recall; Simple and Complex Finger Tapping; DSST; Continuous Performance Task (CPT); Serial Sevens; Positive and Negative Affect Scale (PANAS—a measure of subjective mood); Contrast Sensitivity; Delayed Word Recall. Immediate Word Recall involved computerized serial presentation of fifteen words in a random order at a rate of one word every 2 s. Following the presentation, participants were required to orally recall the words in any order. Presentation and recall occurred three times. The dependent variable was the mean number of words recalled per trial (averaged of three trials). False positives were not documented (e.g., saying a new word). Delayed Word Recall (45 min postinitial presentation) involved one attempt to orally recall the words presented during Immediate Word Recall. Simple Finger Tapping involved pressing the “2” key as many times as possible with the index finger over 10 s. The forearm was fixed, and two trials were performed with each hand. The first trial was considered a practice, and data were only collected for the second trial. Complex Finger Tapping followed the same procedure expect the required response was 1–2–3 (consecutively) using the forefinger for 1, the second finger for 2 and the third finger for 3. The dependent variable was the total number of responses, averaged across the dominant and non-dominant hand (errors were not recorded). The DSST [19] is a pen and paper test which contains a key of nine digit-symbol pairs and an accompanying list of digits. Under each listed digit, a space is provided for the participant to enter the corresponding symbol. The dependent variable was the number of symbols correctly entered over 90 s. The CPT involved random presentation of letters at a rate of one every 250 ms for a duration of 6 min. Participants were required to press the space bar when a letter other than “X” was presented. The dependent variable was the number of false positives (i.e., pressing the space bar when an “X” appeared). Serial Sevens required participants to orally count down in sevens from a randomly determined starting number ranging from 700 to 1000. The dependent variable was the number of correct responses over 2 min. Correct answers were determined relative to the previous number given, regardless of whether the previous response was correct or incorrect. The PANAS [23] requires a response to twenty adjective items on a scale ranging from 1 to 5 according to how the respondent is feeling at that moment with “not at all” and “extremely” as the anchor points. Scoring is collated into two factors reflecting Positive Affect and Negative Affect. In addition, analysis of each PANAS mood adjective was also performed. The Contrast Sensitivity test required identification of a series of letters on a computer screen. The letters become increasingly difficult to distinguish as the contrast between the letters and the background is reduced. The dependent variable was the point at which the letters could not be identified (Michelson Contrast).

### Statistical analysis

Given the importance of considering baseline cognitive performance prior to drink consumption, change from baseline was calculated at each time point (2 and 6 h) for each cognitive test, and 2 × 2 repeated-measures, Bonferroni-corrected ANOVAs (Drink × Time) were performed using the change from baseline data with planned contrasts (*t* tests) to compare each drink (FR vs placebo) at 2 and 6 h. To examine equality at baseline, *t* tests were performed comparing FR and placebo at baseline for each cognitive test. In addition, for consistency, interpretability and agreement with other studies [24], the cognitive tests were combined to create a measure of global cognitive function. To do this, initially for each cognitive test, the change from baseline data was combined across all drink conditions (placebo and FR) and time points (2 and 6 h), thus forming one variable for each cognitive test which was subsequently converted into *z*-scores [24]. Subsequently, the mean *z*-score for each drink condition (placebo and FR) and each time point (2 and 6 h) was calculated such that global cognitive function = (*Z*_DSST_ + *Z*_Serial Sevens _+ *Z*_Immediate recall_ + *Z*_Delayed recall_ + *Z*_CPT_ + *Z*_simple finger tapping_ + *Z*_complex finger tapping_ + *Z*_Contrast Sensitivity_)/8. The *z*-score data for Contrast Sensitivity and the CPT were multiplied by −1 prior to calculating global cognitive function since a higher score on these two tests indicates worse performance. Repeated-measures 2 × 2 ANOVAs (Drink × Time) were then performed with change from baseline *z*-score data for global cognitive function. Change from baseline was calculated for subjective mood data which were subsequently analyzed with Bonferroni-corrected repeated-measures 2 × 2 (Drink × Time) ANOVAs. Data were analyzed using SPSS Inc. PASW Statistics 18.

## Results

### Cognitive function

**Fig. 1:**
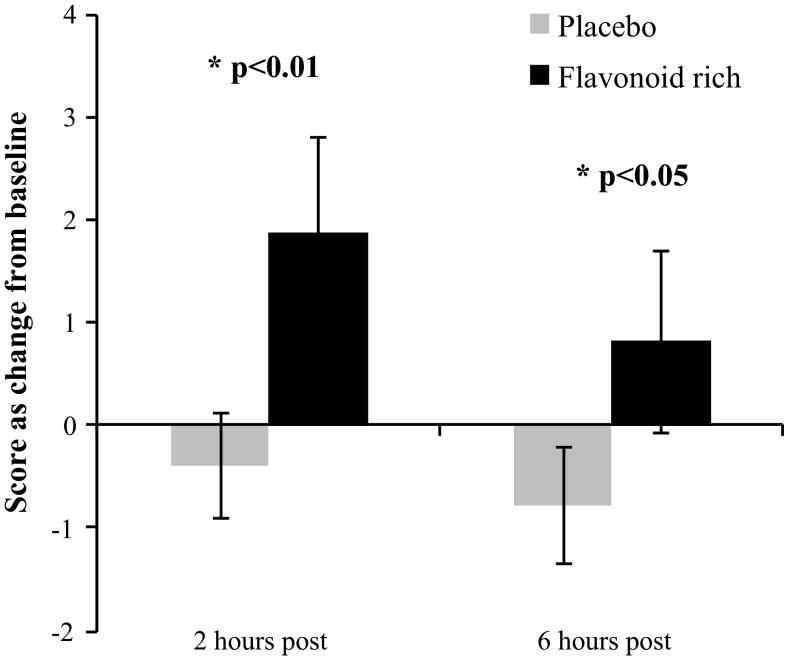
Simple Finger Tapping performance was significantly better following the FR drink relative to the placebo as indicated by a main effect of drink (*F*[1, 20] = 8.32, *p* < 0.01). The change from baseline was significantly higher following the FR drink relative to the placebo at 2 h post consumption and 6 h post consumption. There was no significant difference in baseline performance between the drinks

**Fig. 2:**
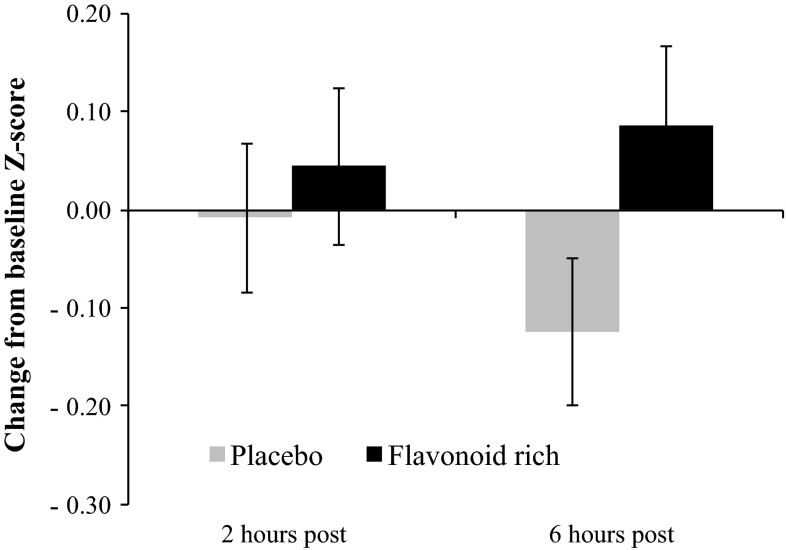
Considering all tests combined, the main effect of drink approached significance (*F*[1,21] = 2.98, *p* = 0.09). At 6 h post consumption, the change from baseline was positive following the FR drink (indicating improvement in global performance), whereas the change from baseline was negative following the placebo (indicating a decline in global performance). The planned contrasts were not performed in light of the nonsignificant ANOVA model. There was no significant difference in baseline performance between the drinks

Raw scores for each cognitive test are shown in Table 2. There were no significant differences between the drinks at baseline for any dependent variables. The 2 × 2 ANOVA revealed a main effect of drink for Simple Finger Tapping (*F*[1, 20] = 8.32, *p* < 0.01) such that performance was better following the FR drink relative to the placebo. More specifically, the mean change from baseline across time points was significantly higher following the FR drink (mean 1.4, SE 0.8) relative to the placebo (mean −0.6, SE 0.5). As shown in Fig. 1, the planned contrasts revealed that the change from baseline was significantly different between the two drinks at 2 h (*t* = 3, *df* = 20, *p* < 0.01) and 6 h (*t* = 2.16, *df* = 20, *p* < 0.05). Similarly, a Drink × Time interaction was observed for CPT accuracy (*F*[1,21] = 3.96, *p* = 0.05) such that change from baseline was significantly different between the two drinks at 6 h (*t* = 2.02, *df* = 21, *p* < 0.05), but no significant difference was observed between the drinks at 2 h. More specifically, following the FR drink, the number of errors was reduced relative to baseline at 6 h (mean −1.6), whereas errors increased following the placebo relative to baseline at 6 h (mean 0.6). Considering all tests combined, the ANOVA model revealed no significant Drink × Time interaction, and no main effect of time. As shown in Fig. 2, the main effect of drink approached significance (*F*[1,21] = 2.98, *p* = 0.09), such that there was a nonsignificant trend for better global cognitive function following the FR drink relative to the control; however, the planned contrasts were not performed in light of the nonsignificant ANOVA model.

**Table 2:** Raw scores per drink condition for each cognitive test and PANAS mood outcomes at all time points (means and SE)

Outcomes measure	Time point	Placebo	Flavonoid-rich orange juice
Digit Symbol Substitution Test (seconds)	Baseline	61.1 (2.3)	62.2 (2.4)
	2 h	61.7 (1.3)	62.9 (2.3)
	6 h	61.6 (2.2)	62.6 (2.2)
Serial Sevens (number correct)	Baseline	39.7 (4.4)	40.9 (4.1)
	2 h	40.1 (4.3)	42 (4.3)
	6 h	40.5 (4.5)	43.5 (4.5)
Immediate Verbal Recall (words)	Baseline	11.1 (.5)	11 (.4)
	2 h	10.2 (.3)	10.2 (.5)
	6 h	10.2 (.4)	10.4 (.5)
Delayed Verbal Recall (words)	Baseline	11 (.7)	11.3 (.6)
	2 h	9.6 (.7)	8.8 (.7)
	6 h	9.5 (.8)	9.4 (.8)
Continuous Performance Task (errors)^a,^*	Baseline	8.2 (1.3)	9.2 (1.6)
	2 h	6.4 (1.2)	7.9 (1.5)
	6 h	8.8 (1.4)	7.6 (1.5)
Simple Finger Tapping* (correct responses)	Baseline	62.1 (1.7)	60.1 (1.8)
	2 h	61.1 (1.7)	61.9 (1.7)
	6 h	61 (1.6)	60.8 (1.7)
Complex Finger Tapping (correct responses)	Baseline	35 (3)	35.7 (3.5)
	2 h	36.1 (2.8)	38 (3.9)
	6 h	33.6 (2)	37.6 (3.9)
Contrast Sensitivity^a^ (Michelson Contrast)	Baseline	3 (.2)	3 (.2)
	2 h	3 (.1)	3.1 (.2)
	6 h	3 (.2)	3 (.2)
Positive Affect (mood) (PANAS scoring)	Baseline	34.1 (1.7)	33.5 (1.7)
	2 h	33.3 (1.6)	33.6 (1.6)
	6 h	31.7 (1.6)	32.5 (1.9)
Negative Affect (mood) (PANAS scoring)	Baseline	11.2 (.4)	12 (.8)
	2 h	10.7 (.3)	10.5 (.3)
	6 h	10.7 (.3)	11.1 (.4)

^a^Higher score indicates worse performance

* A significant main effect of drink was observed for Simple Finger Tapping following a 2 × 2 repeated-measures ANOVA with the change from baseline data, such that performance was following the FR drink relative to the placebo (*p* < 0.05). A Drink × Time interaction was observed for CPT accuracy such that change from baseline was significantly different between the two drinks at 6 h (*p* < 0.05)

### Subjective mood

**Fig. 3:**
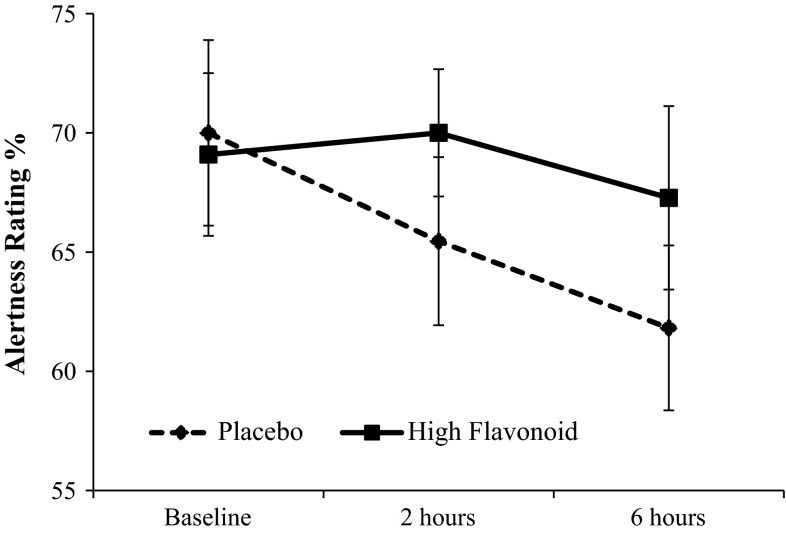
Ratings of subjective alertness using the PANAS were significantly higher following the FR drink relative to the placebo as indicated by a main effect of drink (*F*[1,21] = 4.1, *p* = 0.05). The change from baseline was significantly higher following the FR drink relative to the placebo when averaged across the time points

There were no significant differences between the FR and placebo drinks for any of the subjective mood ratings at baseline. The 2 × 2 ANOVA revealed a main effect of drink for the mood adjective alertness (“how alert do you feel now”) (*F*[1,21] = 4.1, *p* = 0.05) indicating that the change from baseline was significantly different between the drinks. As shown in Fig. 3, a 6.4 % decline (SE 1.8) in alertness was observed over the morning relative to baseline following the placebo, whereas a 0.5 % decline (SE 2.7) was observed following the FR drink relative to baseline. This indicates that the FR drink attenuated a decline in alertness which was observed following the placebo. The individual planned contrasts comparing change from baseline alertness ratings between the drinks did not reach significance at either 2 or 6 h post consumption. Raw scores for Positive Affect and Negative Affect are shown in Table 2. The 2 × 2 ANOVA revealed no significant main effects or interactions for Positive Affect or Negative Affect.

## Discussion

Consumption of FR orange juice was associated with improvements in cognitive function and subjective alertness relative to an energy-matched placebo in healthy middle-aged adults free of disease or mild cognitive impairment. Specifically, scores for the CPT (a measure of attention and more broadly executive function) were significantly better 6 h post orange juice consumption relative to the placebo, while finger tapping (a measure of psychomotor speed) was significantly better 2 and 6 h post consumption relative to the placebo. These findings were reflected by a nonsignificant trend showing improvement in global performance (all tests combined) throughout the day following the orange juice relative to baseline, in contrast to a subtle decline in global performance at both time points relative to baseline following the placebo. These effects on objective measures of cognitive function were mirrored by subjective alertness ratings whereby orange juice consumption attenuated a decline in alertness at both time points following the placebo. This indicates that FR orange juice can enhance objective and subjective cognitive function acutely overly 6 h.

It is important to acknowledge that orange juice consumption was not associated with a significant improvement on every individual cognitive test; this would not be expected given that the effects of nutritional interventions on cognitive performance are small and difficult to detect in healthy adults [25]. However, consistently higher means were observed following orange juice relative to the placebo in the vast majority of outcomes (see Table 2). This is consistent with the findings from an 8-week chronic orange juice intervention in healthy older adults which showed improved global cognitive function relative to a low-flavonoid control [11]. This emphasizes the importance of considering cognitive function as a whole rather than focusing exclusively on individual cognitive tests and domains. Nevertheless, it is interesting to note that the strongest benefits of orange juice were observed for the CPT and Simple Finger Tapping, tasks which required strong elements of executive function and psychomotor speed, respectively. Kean et al. [11] also reported subtle improvements for executive function following dietary supplementation of flavanone-rich orange juice; however, other chronic studies have focused almost exclusively on memory. For example, 12–16 weeks of grape or blueberry juice consumption has been shown to benefit memory in adults with mild cognitive impairment, but executive function was not assessed [8–10].

A key finding from the present research was the observation that cognitive benefits following a FR drink can occur within the immediate 6-h postprandial time period. Hendrickson and Mattes [26] failed to find any acute effects when cognitive performance in young adults was assessed in the afternoon following consumption of a grape juice drink during lunch. The younger sample recruited by Hendrickson and Mattes [26] (mean age 26) could account for the null effects relative to the present study (mean age 51); it is conceivable that healthy young adults typically perform at peak cognitive capability, and therefore, nutritional interventions may have little scope for impact. Having said that, benefits for executive function [15], spatial working memory and reaction time [14] have been observed in healthy young adults 2 h post consumption of non-fruit-based flavanol-rich cocoa drinks. Furthermore, general increases in mental fatigue over the morning were attenuated by a 520-mg cocoa flavanol drink [15], which reflects the attenuation of a decline in subjective alertness following the FR drink in the present study. Collectively, these data indicate that flavonoid-based acute nutritional interventions can improve performance on cognitive tests and enhance subjective mood and alertness in healthy adults. This highlights the possibility that cognitive effects are mediated by subjective mood, and proposed mechanisms of action for flavonoids on the brain should consider this.

Examining the potential underlying mechanisms was not the aim of this research; however, the greatest cognitive benefits were observed at 6 h which is consistent with the anticipated peak in plasma flavanone metabolites [21, 22]. In support, pilot bioavailability data (*n* = 6) in healthy adults following consumption of the same FR drink showed peak concentrations of 0.25 µM hesperetin and 0.12 µM naringenin in the plasma at 6 h. Therefore, it is reasonable to propose that mechanisms relating to the flavanones hesperidin and narirutin may underlie the cognitive benefits at 6 h. This hypothesis requires investigation as plasma flavanone concentration was not measured concomitantly with the behavioral outcomes. Effects at 2 h are more difficult to explain via this mechanism given that the concentration of metabolites would not be expected to be peak at this point according to the timescale for these flavanones to be absorbed into the small intestine, converted into glycosides, hydrolyzed by microflora in the large intestine, and appear as glucuronidated and sulfated metabolites in the plasma [22, 27, 28]. However, aforementioned research with cocoa shows that behavioral effects can be achieved within the 2-h postprandial period following flavonoid consumption [14, 15]. It is possible that the flavanone metabolites may be exerting effects in advance of reaching peak concentration in the plasma.

Once ingested and metabolized, the specific mechanisms by which flavonoids may affect cognitive function have been described in detail elsewhere [29, 30]. Flavanone metabolites are known to cross the blood-brain barrier following oral ingestion [31], and research on rodents indicates that flavanones may have specific neuroprotective effects such as increasing the expression of brain-derived neurotrophic factor (BDNF) [32, 33]. However, it is unlikely mechanisms relating to BDNF and synthesis of proteins and enzymes in the brain such as cAMP-response element-binding protein (CREB), which affect neuronal signal transduction [34], could be effective within a 6-h timescale. One plausible hypothesis is that flavonoid consumption may lead to acute cognitive benefits via increased CBF as a result of enhanced endothelial function and increased bioavailability of nitric oxide [35, 36]. Human studies show significantly increased CBF several hours following cocoa flavanol consumption [12, 36]. These are supported by chronic studies which show increased activation in the right middle prefrontal cortex and the right superior parietal cortex following anthocyanin and flavanol-rich grape juice consumption [9]. Moreover, increased steady-state-evoked potentials in posterior parietal and central–frontal regions [37] and increased CBF in the hippocampus during a spatial memory task [13] have been observed following several weeks daily consumption of cocoa flavanols. As yet, there are no published data examining peripheral or CBF in humans following flavanone consumption; therefore, these potential mechanisms are speculative at this time.

It is possible that the observed cognitive effects in this study could be in part accounted for by vitamin C and/or folate which were not matched across the FR and placebo drinks. Cognitive benefits associated with these micronutrients are only likely to occur following chronic consumption over several weeks and months, or in cases where there are deficiencies [4]. Therefore, it is unlikely that vitamins or folate could account for the cognitive benefits observed 2–6 h post consumption in this healthy sample. A strength of the present study is that the beneficial effects occurred within the everyday context of consuming a standardized breakfast and lunch, indicating that positive effects are not dependent upon a period of fasting and relative energy deprivation. To extrapolate, FR orange juice-based interventions can offer acute cognitive benefits over the course of the day when consumed in conjunction with normal dietary intake. Having said that, participants were instructed to consume a low-flavonoid diet prior to each test day; therefore, it is unclear whether a habitually high-flavonoid diet would mask the effects of similar interventions. With regard to the generalizability of these findings, the orange juice drink was a commercial available product (Tropicana Pure Premium Orange Juice) with the addition of FR pomace, which is an edible part of the whole orange usually leftover during the production of the juice (see method). This FR drink is not currently available to the public although it is important to point out that it consists entirely of edible oranges.

To conclude, consumption of an orange juice containing 272 mg flavonoids was associated with acute improvements in cognitive function and subjective alertness up to 6 h post consumption relative to an energy-matched control drink containing no flavonoids in healthy middle-aged adults. Fruit juices and their respective fruits are an easily available commonly consumed source of flavonoids and polyphenols. These data demonstrate that fruit juice-based flavonoids can acutely enhance cognition in healthy adults. This is consistent with the accumulating evidence from chronic interventions and epidemiological research that increased consumption of fruits, fruit juices and other flavonoid-rich foods over the lifespan is associated with cognitive benefits such as a reduced risk of neuropsychological disease, attenuation of aging-induced cognitive decline and maintenance of optimal cognitive facilities [4].
